# Mechanisms Underlying Early Rapid Increases in Creatinine in Paraquat Poisoning

**DOI:** 10.1371/journal.pone.0122357

**Published:** 2015-03-27

**Authors:** Fahim Mohamed, Zoltan Endre, Shaluka Jayamanne, Timothy Pianta, Philip Peake, Chathura Palangasinghe, Umesh Chathuranga, Kithsiri Jayasekera, Klintean Wunnapuk, Fathima Shihana, Seyed Shahmy, Nicholas Buckley

**Affiliations:** 1 South Asian Clinical Toxicology Research Collaboration, University of Peradeniya, Peradeniya, Sri Lanka; 2 Clinical Pharmacology and Toxicology Group, Professorial Medicine Unit, The Prince of Wales Clinical School, University of New South Wales, Sydney, Australia; 3 Department of Pharmacy, Faculty of Allied Health Sciences, University of Peradeniya, Peradeniya, Sri Lanka; 4 Department of Nephrology, Prince Of Wales Hospital and Clinical School, University of New South Wales, Sydney, Australia; 5 Therapeutics Research Centre, School of Medicine, University of Queensland, Brisbane, Australia; 6 Department of Pharmacology, School of Medical Sciences, Sydney Medical School, University of Sydney, Sydney, Australia; Anatomy, SWITZERLAND

## Abstract

**Background:**

Acute kidney injury (AKI) is common after severe paraquat poisoning and usually heralds a fatal outcome. The rapid large increases in serum creatinine (Cr) exceed that which can be explained by creatinine kinetics based on loss of glomerular filtration rate (GFR).

**Methods and Findings:**

This prospective multi-centre study compared the kinetics of two surrogate markers of GFR, serum creatinine and serum cystatin C (CysC), following paraquat poisoning to understand and assess renal functional loss after paraquat poisoning. Sixty-six acute paraquat poisoning patients admitted to medical units of five hospitals were included. Relative changes in creatinine and CysC were monitored in serial blood and urine samples, and influences of non-renal factors were also studied.

**Results:**

Forty-eight of 66 patients developed AKI (AKIN criteria), with 37 (56%) developing moderate to severe AKI (AKIN stage 2 or 3). The 37 patients showed rapid increases in creatinine of >100% within 24 hours, >200% within 48 hours and >300% by 72 hours and 17 of the 37 died. CysC concentration increased by 50% at 24 hours in the same 37 patients and then remained constant. The creatinine/CysC ratio increased 8 fold over 72 hours. There was a modest fall in urinary creatinine and serum/urine creatinine ratios and a moderate increase in urinary paraquat during first three days.

**Conclusion:**

Loss of renal function contributes modestly to the large increases in creatinine following paraquat poisoning. The rapid rise in serum creatinine most probably represents increased production of creatine and creatinine to meet the energy demand following severe oxidative stress. Minor contributions include increased cyclisation of creatine to creatinine because of acidosis and competitive or non-competitive inhibition of creatinine secretion. Creatinine is not a good marker of renal functional loss after paraquat poisoning and renal injury should be evaluated using more specific biomarkers of renal injury.

## Introduction

Deliberate self-poisoning with paraquat, a non-selective contact herbicide, is a major public health problem in many countries in South East Asia [1,2]. Case fatality is between 40–60% [3,4] and death usually occurs within 24–72 hours of ingestion in severe poisoning [4]. No current treatment strategy is effective [4]. While a range of biochemical and clinical prognostic markers have been investigated to identify early organ damage and predict outcomes [5], there is currently no satisfactory marker for associated kidney injury even though this is one of the most prominent clinical features [3,6,7] and an important predictor of death [7,8]. Paraquat accumulates inside renal tubular cells causing reduction-oxidation (redox) cycling and increases reactive oxygen species formation with subsequent proximal tubular injury [9]. A decrease in paraquat elimination due to renal failure aggravates systemic toxicity [10]. AKI is currently defined by either change in creatinine or urine output [11,12] despite inherent limitations of creatinine in sensitivity and specificity to AKI. Changes in these traditional biomarkers may involve a substantial delay and both may be altered by various non-renal factors [13].

Creatinine is the most commonly studied marker of renal injury in paraquat poisoning [3,6,7]. Rapid changes in serum creatinine from the baseline (>100%) within 12–24 hours of ingestion have been reported in some small studies [3,6,14]. Such rapid changes in creatinine are difficult to explain solely by direct nephrotoxic loss of glomerular filtration [15], and raise the possibility that non-renal mechanisms or assay-based artefacts have contributed to these results [16–18]. A better understanding of reasons for rapid changes in serum creatinine may help guide emergency management.

We hypothesized that rapid elevation of creatinine within 24 hours following paraquat poisoning was not solely due to paraquat-induced AKI. We assessed this prospectively by comparing the kinetics of two surrogate markers of GFR, serum creatinine and serum cystatin C (sCysC), in order to understand and assess renal functional loss after severe paraquat poisoning in a large multi-centre study.

## Materials and Methods

### Study Design and Data Collection

Consenting patients were part of a multi-centre prospective cohort study of adult poisoning patients admitted to five hospitals in Sri Lanka. Paraquat ingestion was confirmed by a positive paraquat urine dithionate test (urine colour changed to blue). The study was approved by the human research ethics committees of the University of Peradeniya and University of New South Wales. Informed written consent was obtained from all patients included in the study. All patients received standard care according to local hospital management guidelines.

Demographic and clinical data were obtained during the hospital admission. The medical records were reviewed to identify all drugs administered as part of medical care including possible nephrotoxins. Hospital biochemistry results were recorded whenever available and additional urinary and plasma samples were obtained. Strict monitoring of urine output was not routine in the study hospitals, so urine output data was only available in a limited number of subjects.

Blood and urine samples were collected at 4, 8, 16 and 24 hours following ingestion and then daily until discharge where possible. All samples were immediately processed, stored briefly at -20°C and subsequently transferred to -70°C until analysis. Assays were conducted in research laboratories in both Sri Lanka and Australia. Serum and urine creatinine concentrations were measure by a modified Jaffe method (kinetic Jaffe reaction method, rate blank and compensate), using Roche Hitachi 912 automatic analysers following the manufacturer’s recommendations. Creatinine assays were repeated in another independent laboratory (Sydney, Australia) on a Konelab^TM^ 20XT clinical chemistry analyser (Thermo Scientific) and analysed using both Jaffe and enzymatic methods since the Jaffe reaction may be affected by circulating non-creatinine chromogens. Serum creatine kinase (CK) and CysC were also quantified in the same plasma samples (Konelab^TM^ 20XT). CysC was measured using a microparticle-enhanced immunoturbidimetric method, according to the manufacturer’s recommendations. Inter and intra assay precisions were less than 10% for both creatinine and CysC assays. Urinary CysC was quantified using Bio-Plex Pro RBM Human Kidney Toxicity Assays panel 2 on the Bio-Plex 200 system (BIO-RAD). Inter and intra assay precisions were <15% and 5% respectively. Paraquat levels were measured at the Therapeutic Research Centre, University of Queensland, Brisbane, Australia using LC–MS/MS as described elsewhere [19].

### Definition of End Points

AKI was diagnosed using the Acute Kidney Injury Network (AKIN) criteria [12]. AKIN Stage I was defined as an increase in serum creatinine to > 150% of baseline or an absolute increase of ≥ 0.3 mg/dl from baseline at any time within 48 hours; AKIN Stage II as an increase of > 200% of baseline, and a creatinine level > 300% of baseline or increase of > 4 mg/dl and an acute rise of at least 0.5 mg/dl as stage III. In the absence of a baseline creatinine measurement within three months prior to injury, the Acute Dialysis Quality Initiative (ADQI) and Kidney Disease Improving Global Outcomes (KDIGO) groups have recommended estimating the baseline serum creatinine by solving the Modification of Diet in Renal Disease (MDRD) equation for a glomerular filtration rate of 75 mL/min/1.73m^2^ (MDRD75) [11], although this introduces systematic errors, mostly overestimating the incidence of AKI [20]. Since, baseline data were not available in the study population, the baseline creatinine was estimated using the following hierarchy: the lowest creatinine value acquired either after follow-up (in those who completed follow-up), or prior to hospital discharge (in those patients who did not complete follow-up), and in non-survivors, whose serum creatinine had not returned to normal levels, the MDRD75 value. A sensitivity analysis was conducted to assess the effect of using an estimated baseline in non-survivors equal to the 75^th^ centiles of estimated baseline values for survivors. Similarly, the lowest sCysC concentration acquired at follow-up or prior to hospital discharge was used as the baseline. A sensitivity analysis in non-survivors was also conducted based on estimated CysC concentrations by solving sCysC equations for a GFR of 75 mL/min/1.73m^2^ [Chronic Kidney Disease Epidemiology Collaboration (CKD-EPI 75) [21].

Serum biomarker concentrations were reported as absolute concentrations [22]. Relative changes in biomarkers were calculated as a percentage in relation to the measured or estimated baseline values. The non-steady state Jelliffe formula [23] was used for the estimation of GFR based on serum creatinine levels. A non-steady state sCysC based GFR formula [24] was also employed to estimate GFR. Paraquat clearance was measured from urine (U_p_) and plasma paraquat (P_p_) concentrations using the formula U_p_*V/P_p,_ where V is urine volume (ml).

### Statistical Analysis

Biomarker and other continuous variable concentrations were reported as median values and interquartile ranges and analysed using the nonparametric Mann-Whitney U test. The Fisher’s exact test was used to compare categorical variables. Spearman’s correlation coefficient was calculated to compare different creatinine assays and exclude chromogen interference. Statistical comparisons were made between AKI/no-AKI and survivors/non-survivors. Descriptive statistics and graphs were generated using GraphPad Prism version 6 (GraphPad Software, San Diego, USA).

## Results

**Fig 1:**
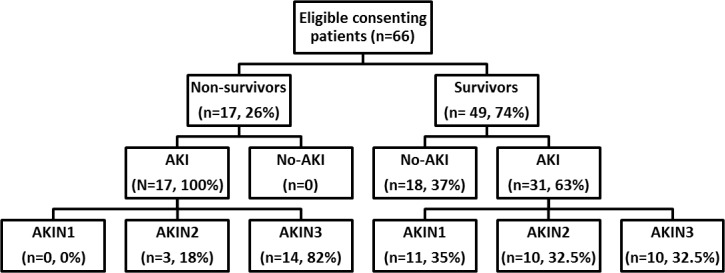
Patient recruitment to cohort study.

**Fig 2:**
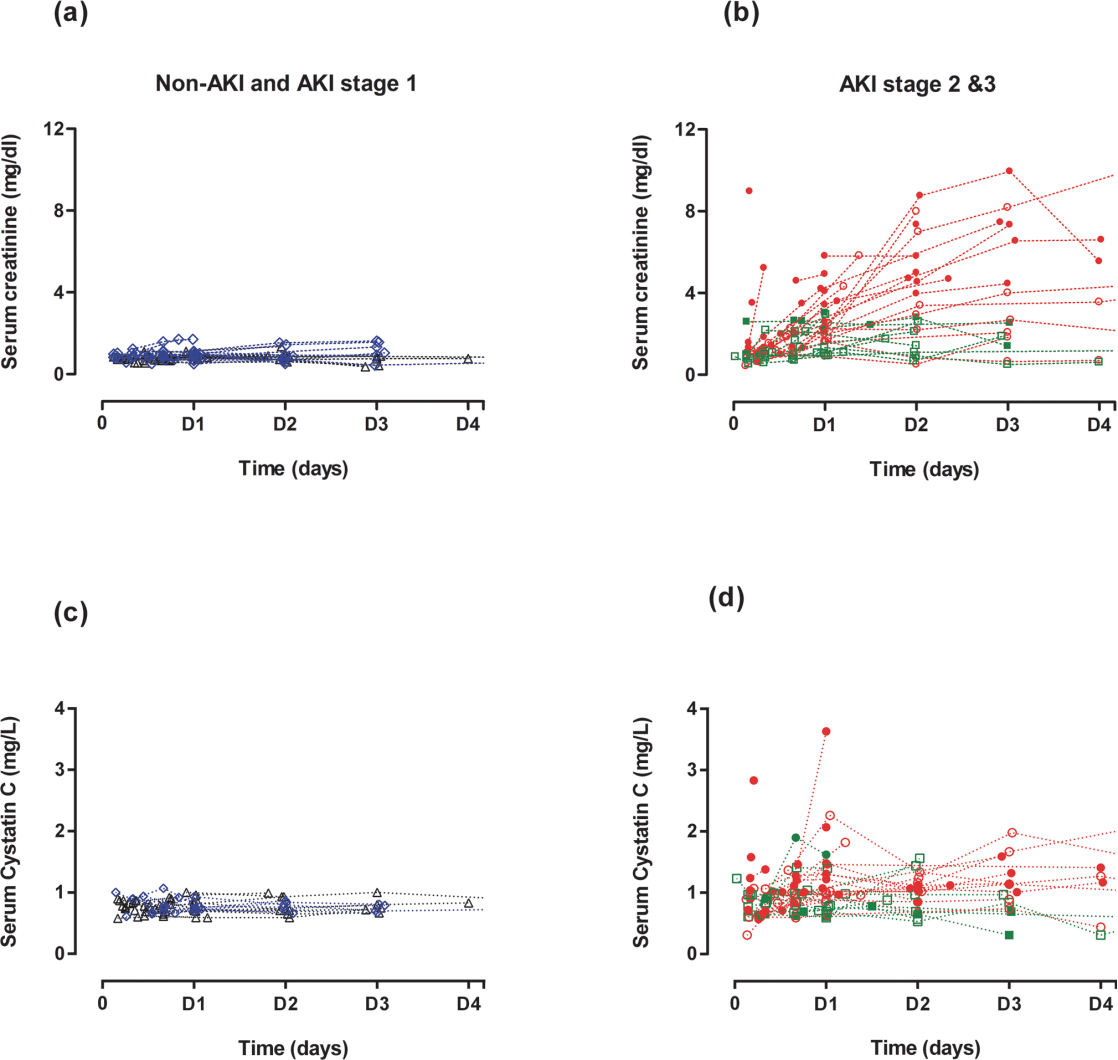
Serial serum concentrations of creatinine and cystatin C relative to AKI and hospital discharge status. This figure represents the changes in absolute creatinine (a & b) and cystatin C (c & d) concentrations in each patients over 4 days following paraquat ingestion. Filled symbols represent patients who died in the hospital and the open symbols represent survivors. Patients were also grouped according to AKI severity; No-AKI (black triangle), AKIN1 (blue rhombus), AKIN2 (green square) and AKIN3 (red circles).

Sixty six (n = 66) patients were available for evaluation and 48 developed AKI. Of patients with AKI, 37 developed moderate to severe AKI (AKIN stage 2 and 3) and seventeen (n = 17) of these died in the hospital. Development of AKI was associated with death in the entire cohort (n = 66 patients, RR = 1.5, OR = 20, p<0.005) (Fig 1). Limited renal replacement therapy was available for the entire hospital and was not offered to patients following paraquat or other herbicide poisonings. Patients were predominantly young, previously healthy males with similar baseline demographic and clinical variables regardless of outcome except for baseline serum creatinine and estimated GFR (Table 1). Estimated baseline serum creatinine was higher in non-survivors, which probably reflects an over estimate in these young patients since creatinine was back-calculated using MDRD75 in all non-survivors [11]. In both survivors and non-survivors, AKI was generally moderate to severe (stage 2 or 3) with rapid increases in serum creatinine concentrations observed in half of the cohort (n = 37). In contrast, creatinine concentration remained relatively unchanged or gradually increased in less severe AKI groups (AKIN1) and in non-AKI (Fig 2). Further analysis was restricted to the 37 moderate to severe AKI cases to explore the causal factors accounting for the observed rapid rise in creatinine in these patients and also observed in similar studies [3,6,14].

**Table 1:** Baseline demographic and clinical profile.

**Baseline characteristics**	**Survivors (n = 49)**	**Non-survivors (n = 17)**
Age (years)	26 (19–31)	32 (19–52)
Male gender (%)	61%	72%
Weight (kg)	50 (45–58)	52 (44–63)
Amount ingested (ml)	20 (10–35)	20 (20–250)
Time to admission from ingestion (hours)	3.2 (1.7–7.0)	4.2 (2.1–11.0)
Pulse (beats/minutes)	80 (80–88)	82 (77–90)
BP systolic (mm Hg)	115 (110–120)	115 (108–122)
BP diastolic (mm Hg)	70 (70–80)	80 (70–80)
Serum creatinine (mg/dl)	0.7 (0.6–0.9)	1.4 (1.2–1.5)*
Serum Cystatin C (mg/l)	0.7 (0.6–0.8)	0.8 (0.7–0.9)
eGFR (ml/min)^£^	102 (75–138)	55 (48–66)*
eGFR (ml/min)^$^	112 (87–124)	113 (103–123)
Time to AKIN1 (hours)	19 (16–29)	16 (9–24)
Time to AKIN2 (hours)	24 (16–46)	24 (16–48)
Time to AKIN3 (hours)	40 (24–55)	35 (14–48)

*Data presented as median and interquartile range for continuous and percentage for categorical variables*. * *represents p<0*.*0001*, *eGFR was estimated from both baseline serum creatinine* ^*£*^ *[25] and cystatin C* ^*$*^ *[21]concentrations.*

### Absolute serum creatinine and cystatin C

In all AKIN 2 & 3 patients, irrespective of subsequent survival, serum creatinine increased rapidly within 16 hours, and continued to rise for at least 48 hours (a 3-fold increase); levels did not return to steady state in non-survivors (Fig 2B & Table 2). In contrast, serum and urinary CysC increased slowly until 24 hours in the same patients and slowly decreased towards steady state levels (Fig 2D & Table 2).

**Table 2:** Serum/urine levels of creatinine and cystatin C.

**Time post-ingestion**	**Absolute creatinine (mg/dl)**	**Change in creatinine (mg/dl)**	**Urinary creatinine (mg/dl)**	**Absolute cystatin C (mg/l)**	**Change in cystatin C (mg/l)**	**Urinary cystatin C (ng/ml)**
4 hours All Survivors Non-Survivors	0.98 (0.85–1.48) 0.87 (0.64–1.0) 1.22 (0.98–2.81)	0.06 (0–0.49) 0.03 (0–0.36) 0.06 (0–1.43)	72 (51–94) 87 (62–264) 51 (41–82)	0.90 (0.63–1.06) 0.95 (0.61–1.0) 0.81 (0.63–1.33)	0.02 (0–0.34) 0.12 (0–0.46) 0.01 (0–0.22)	50.5 (23.6–101.2) 49.9 (20.3–62.6) 69.0 (24.7–197.9)
8 hours All Survivors Non-Survivors	1.07 (0.88–1.37) 0.72 (0.57–0.99) 1.28 (1.02–1.73)	0.05 (0–0.54) 0.05 (0–0.65) 0.05 (0–0.43)	65 (43–165) 124 (42–206) 60 (28–75)	0.83 (0.66–0.99) 0.83 (0.66–0.97) 0.71 (0.66–1.02)	0.05 (0–0.16) 0.06 (0–0.28) 0.03 (0–0.14)	32.6 (8.7–57.8) 35.6 (27.3–57.0) 15.4 (5.7–132.2)
16 hours All Survivors Non-Survivors	1.46 (1.11–2.08) 1.22 (1.0–1.88) 2.0 (1.34–2.84)	0.57 (0.24–1.04) 0.57 (0.48–0.84) 0.6 (0–1.77)	52 (36–120) 86 (33–174) 42 (30–76)	0.98 (0.78–1.20) 0.85 (0.78–1.00) 1.06 (0.80–1.33)	0.25 (0.01–0.38) 0.25 (0.01–0.38) 0.27 (0–0.36)	117.8 (31.5–233.4) 101.9 (8.9–272.3) 138.2 (51.8–211.5)
24 hours All Survivors Non-Survivors	2.16 (1.54–3.13) 1.61 (1.05–2.16) 3.03 (2.30–4.14)	1.1 (0.64–1.84) 0.84 (0.38–1.43) 1.66 (1.0–2.8)	46 (26–119) 72 (16–164) 38 (26–76)	1.01 (0.79–1.43) 0.92 (0.72–1.03) 1.3 (1.0–1.55)	0.31 (0.14–0.6) 0.21 (0.05–0.45) 0.37 (0.26–0.81)	103.3 (28.5–254.0) 89.8 (23.7–132.3) 156.6 (73.5–283.1)
48 hours All Survivors Non-Survivors	2.93 (1.58–3.48) 2.11 (0.92–2.38) 4.70 (3.67–6.18)	2.4 (0.82–3.78) 1.35 (0.43–2.42) 3.51 (2.76–6.14)	58 (50–101) 70 (52–120) 54 (24–57)	1.00 (0.71–1.12) 0.97 (0.69–1.25) 1.02 (0.74–1.09)	0.2 (0–0.41) 0.23 (0.03–0.42) 0.03 (0–0.42)	115 (48.8–204) 52 (36.5–168) 205 (115.0–608)

*Absolute creatinine in severe patients increased 3 fold over next 48 hours and the levels continue to increase*. *However*, *both serum and urinary cystatin C levels remain steady over time*. *Change in creatinine and cystatin C represent the absolute change in these two functional markers from the baseline values*. *Data are presented as median with IQR*.

### Relative change in creatinine and CysC

**Fig 3:**
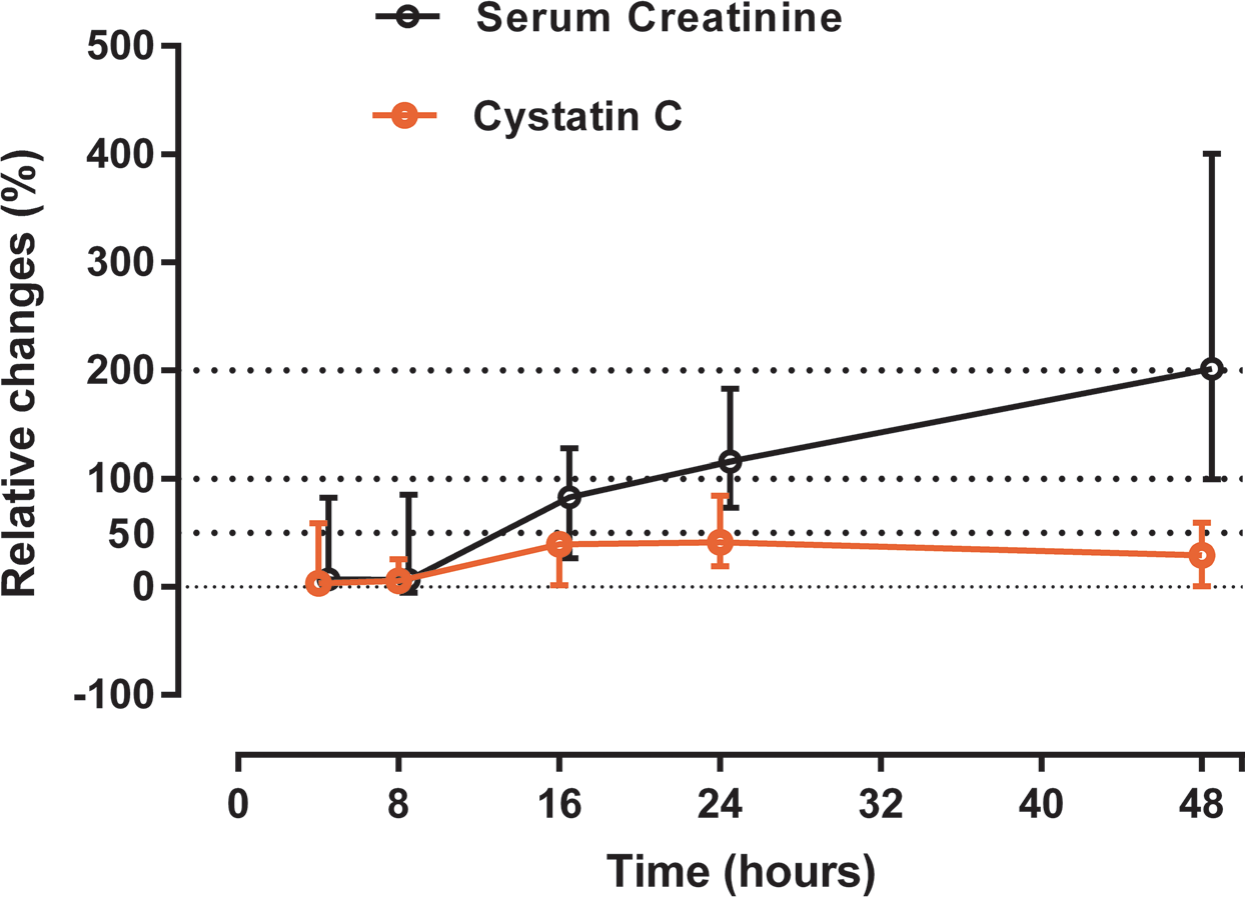
Relative changes (%) in both creatinine and cystatin C. Total of 37 severe patients included in this graph (non-survivors = 17 patients). In all the survivors, baseline was assumed as lowest concentrations during the hospital stay or at follow up (for both serum creatinine and serum cystatin C). Baseline serum creatinine level in non-survivors was estimated by solving MDRD formula for GFR of 75 ml/min.

**Fig 4:**
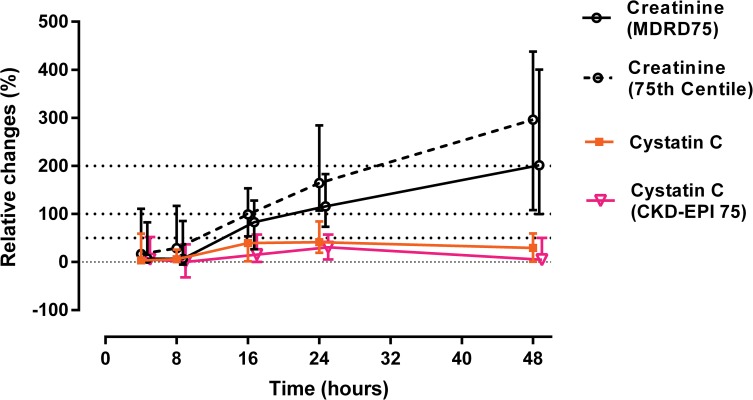
Relative changes (%) in both creatinine and cystatin C based different spectrum of baseline estimates. Total of 37 severe patients included in this graph (non-survivors = 17 patients). In all the survivors, baseline was assumed as lowest concentrations during the hospital stay or at follow up. Baseline serum creatinine level in non-survivors was estimated by solving MDRD formula for GFR of 75 ml/min [black bolded line in the graph- serum creatinine (MDRD75)] or 0.9 mg/dl (75% of survivors had baseline levels below 0.9 mg/dl) (black dotted line- serum creatinine (75th centile). Similarly, baseline serum cystatin C in non-survivors was assumed as lowest values obtained (orange line) or estimated by solving cystatin C based CKD-EPI formulas (pink line) 21 for GFR of 75 ml/min [133 × (Cystatin C /0.8)−1.328 × 0.996Age (× 0.932 if female)].

In patients with higher severity AKI (AKIN 2 & 3) following paraquat, there was a 100% increase in median creatinine relative to baseline by 24 hours and 200% by 48 hours (Fig 3). The increase in creatinine was 300% relative to baseline by 72 hours. Because these changes were based largely on estimated baseline creatinine values, a sensitivity analysis was conducted to assess the effect of using different baseline assumptions for both serum creatinine and CysC. Seventy five percent (75%) of survivors had baseline creatinine levels below 0.9 mg/dl. Based on demographic similarity to survivors, the baseline level of non-survivors could be assumed to be 0.9 mg/dl. Using this estimate an even greater than 300% increase in creatinine was seen at 48 hours, and the apparent disparity between creatinine and CysC increased (Fig 4).

In contrast to creatinine, there was a minimal increase in sCysC over the first 24 hours. A 50% increase in CysC occurred by 24 hours post ingestion, which remained largely unchanged up to 72 hours (Fig 3). Consequently there was a progressively increasing gap between the relative increase in CysC and creatinine. This disparity amounted to a 2, 3, 6 and 8 fold increase in creatinine relative to CysC at 16, 24, 48 and 72 hours respectively. In addition, creatinine and CysC concentrations were weakly correlated (r = 0.1, p >0.05) in those patients who didn’t develop AKI following paraquat poisoning.

### Change in other kidney functional indices and urinary paraquat concentration

**Fig 5:**
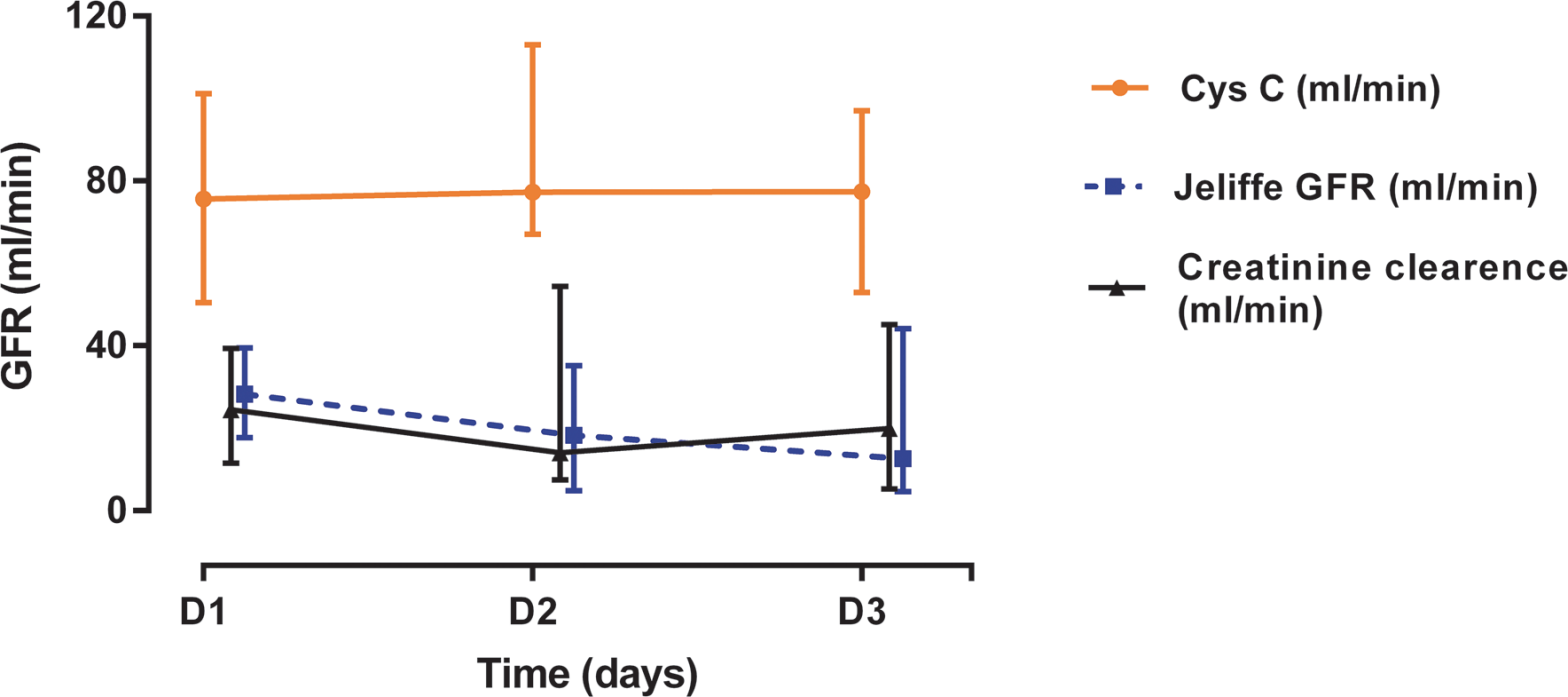
Daily GFR estimates in paraquat patients. GFR was estimated based on serum cystatin C and creatinine and demonstrated a twofold higher GFR for estimation based on cystatin C estimates than creatinine.

Other urinary functional indices such as urinary albumin, and total protein remained steady during the period that serum creatinine rose rapidly (Table 3). Urinary paraquat concentrations were initially high for 24 hours then declined (Table 3). By 24 hours the median paraquat clearance was estimated to be 31 ml/min (IQR 15 to 61), which was slightly higher than the estimated apparent creatinine clearance (Fig 5). Urine output data was available for a subgroup of 12 patients in the AKI group. Two patients in this group who died were anuric, but most had a urine output of greater than 0.75 ml/kg/hour. The median urine output at 24 hours in AKI group was 0.68 (IQR 0.46–1.25) ml/kg/hour, and this value was used to estimate both creatinine and paraquat clearance in patients in whom urine volume data were missing.

**Table 3:** Other kidney function indices profiles.

**Time post-ingestion (hours)**	**Serum Albumin (g/dl)**	**Serum total protein (g/dl)**	**Plasma urine creatinine ratio**	**Urine paraquat (mg/L)**
4	4.4 (3.2–4.7)	7.2 (6–8.2)	0.02 (0–0.02)	5.2 (2.3–54)
8	4.5 (4–4.7)	6.8 (6.1–7.8)	0.02 (0–0.02)	28.4 (8.4–185)
16	4.1 (3.3–4.7)	6.6 (6–7.6)	0.02 (0.01–0.05)	11.8 (6.7–48.6)
24	4 (3.4–4.7)	6.7 (6.1–7.6)	0.03 (0.01–0.06)	5.6 (2.2–24)
48	4 (3.1–4.3)	6.7 (6.1–7.3)	0.04 (0.01–0.08)	3.0 (2.3–4.7)

*These urinary indices didn’t change significantly over 48 hours of the injury*. *Data are presented as median and IQR*. *This table is based on data obtained from 37 patients who developed AKI 2 &3*.

### Estimated Glomerular Filtration Rate (GFR) based on creatinine and CysC concentrations

The estimated non-steady state GFR based on CysC level was at least 2 fold higher each day compared to the creatinine based non-steady state GFR estimates or the directly estimated creatinine clearance (Fig 5).

### Factors that may alter creatinine and CysC in paraquat poisoning

Plasma paraquat concentration, the method of creatinine assay, and CK activity may each alter the levels of creatinine observed in paraquat poisoning. Hence each of these factors was examined but none provided a satisfactory explanation for the observed early large increases in serum creatinine.

**Fig 6:**
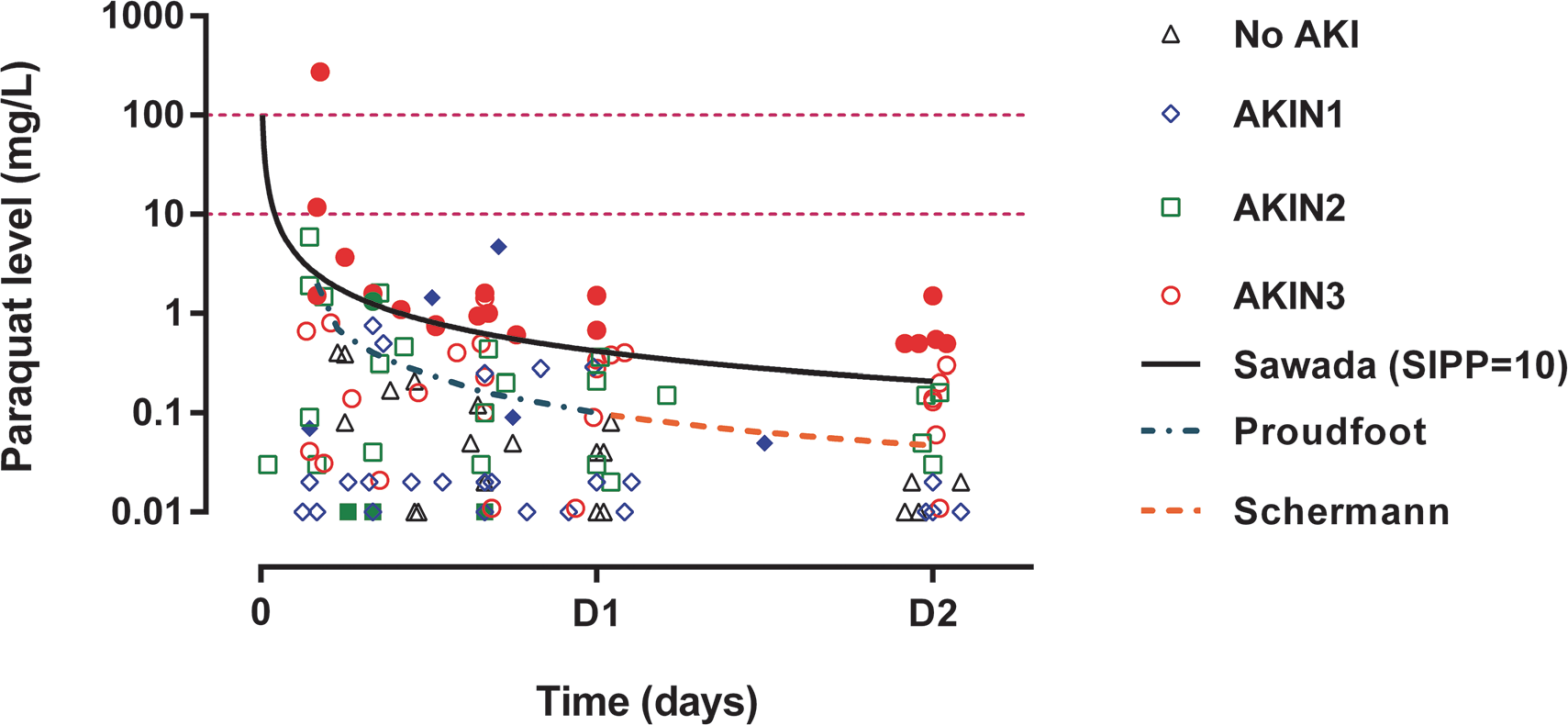
Serial plasma paraquat concentration according to AKI severity and hospital discharge status. This graph displays the predictability of deaths by current paraquat nomograms in this cohort. Filled symbols represent patients who died in the hospital and the open symbols represent survivors.

**Fig 7:**
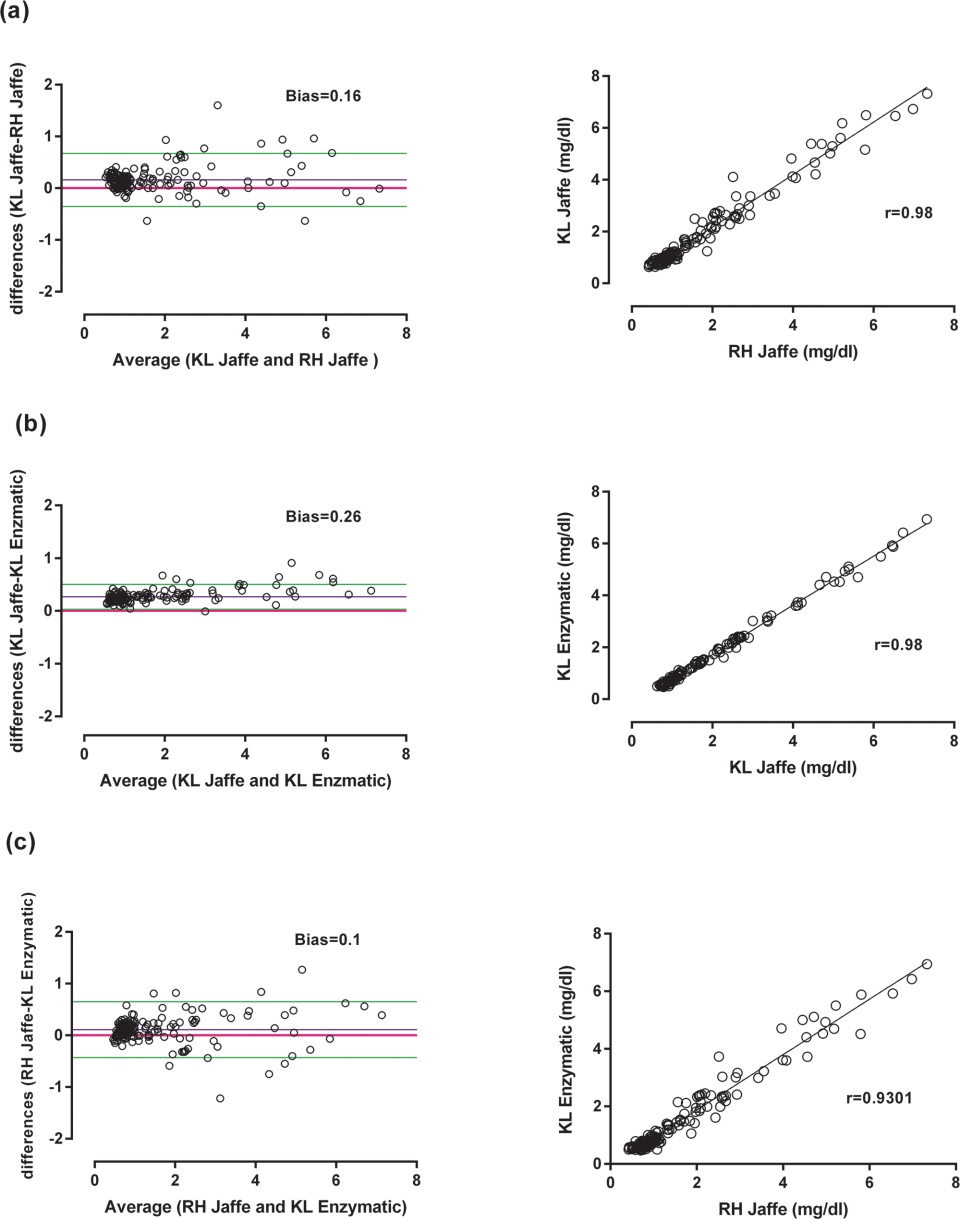
Correlation and Bland-Altman plot for two different creatinine assay methods in two independent laboratories on same samples. Excellent correlation was obtained between these two methods (a) Konelab (KL) Jaffe and Roche Hitachi (RH) Jaffe, (b) Konelab (KL) Jaffe and Konelab (KL) enzymatic and (c) Roche Hitachi (RH) Jaffe and Konelab (KL) enzymatic.

Almost all patients had plasma paraquat concentrations below 10 mg/L and only one had a concentration greater than 100 mg/L. The current nomogram outcome prediction lines predicted death well in this cohort (Fig 6). The creatinine values assayed by both Jaffe methods were strongly correlated (bias 0.16, r = 0.98) (Fig 7A), and there was an excellent correlation also between the Konelab Jaffe and enzymatic method (bias 0.26, r = 0.98) (Fig 7B) and Roche Hitachi Jaffe and the enzymatic method (bias 0.1 and r = 0.93) (Fig 7C).

Only one patient in this cohort had CK levels greater than 1000 units/L. The median CK level was 80 U/L (Range 11–3004, IQR 45–151). Elevated creatinine concentrations didn’t correlate with that of CK in the entire cohort (r = 0.16) or when stratified according to various creatinine concentrations (r = 0.04 at creatinine levels < = 2 mg/dl, r = -0.12 levels between 2 to 4 mg/dl and r = 0.21 at levels greater than 4 mg/dl).

We also explored the possibility of whether circulating paraquat ion suppresses CysC production. Serum CysC concentrations in both AKI and no-AKI group displayed a moderate positive correlation with paraquat concentration (r = 0.3, p<0.005) when compared with serum paraquat concentration suggesting sCysC production is not suppressed by paraquat ions.

## Discussion

This large cohort study of acute paraquat poisoning examined factors that may contribute to a rapid increase in creatinine levels. Overall 73% of the patients developed AKI based on the current consensus definition [11] within 16 and 19 hours respectively in non-survivors and survivors. Similar observations have been reported following acute paraquat poisoning in several studies [3,6,14]. The rise in creatinine was more rapid in severely poisoned [4] patients irrespective of baseline creatinine. Severely poisoned patients showed a relative increase of more than 100% within 24 hours and > 300% by 72 hours post-ingestion, consistent with other studies following severe paraquat poisoning [3,6,14]. Patients in this cohort were previously healthy young adults (Table 1). All non-survivors had an increase in absolute creatinine greater than 2 mg/dl within 24 hours. This is much greater than the maximum possible predicted by creatinine kinetic studies, which suggests an absolute increase of greater than 1.5 mg/dl could only be seen after approximately 48 hours of an acute decrease in GFR assuming a normal baseline [26,27]. Thus these results suggest that an elevated creatinine is a useful prognostic marker of death, but a poor biomarker of renal function in the setting of acute paraquat ingestion. The most likely explanation appears to be increased creatine and creatinine production and mobilisation in response to paraquat-induced oxidative stress.

We first excluded interference in the creatinine assays by non-creatinine chromogens since the creatinine assay is susceptible to interference by non-renal factors such as the concentration of paraquat in body fluids, and assay methods [28], co-administered drugs such as co-formulants of dexamethasone [18], and cephalosporin which may also interfere with creatinine concentrations [17,18,29,30]. The Jaffe serum creatinine assay cross-reacts with plasma paraquat concentrations greater than 10 mg/L, but marked increases were only seen at levels greater than 100 mg/L [16,29]. Only one patient had a paraquat level greater than 100 mg/L concurrent with a creatinine of 8.96 mg/dl. All other samples had concentrations less than 12 mg/L (Fig 6). This indicates that the increase in creatinine concentration was not driven by cross-reactivity with plasma paraquat in these patients. Other non-creatinine chromogens do not interfere with enzymatic methods of measuring creatinine [31]. Since creatinine measured using both methods on the same samples were highly correlated (r = 0.97), the observed early increases in creatinine are unlikely to result from the assay method used.

Paraquat induced rhabdomyolysis is uncommon following moderate to severe paraquat poisoning [4,30,32], although myocytes subjected to paraquat toxicity might release cellular contents including creatine, creatinine and CK [33]. A few studies have reported a late rise in CK around three days after paraquat exposure as a result of accumulation of paraquat in the myocytes [34]. However, creatinine levels didn’t change drastically at that time point [30] and, in any case, increase CK does not correlate well with severity of acute kidney injury [35]. In our cohort, the median CK concentration was only 80 U/L (Range 11–3004, IQR 45–151) and CK levels did not correlate with creatinine suggesting that myocyte injury was not responsible for the observed rapid increase in creatinine.

CysC is a protein molecule (molecular weight 13 kDa) synthesized by all nucleated cells. CysC has the advantage of a shorter half-life [36] compared with creatinine at normal GFR (1–2 vs. 4 hours) [37]. CysC is produced at a constant rate [38], freely filtered by the glomeruli, and normally completely reabsorbed and catabolised by proximal tubule epithelial cells [39,40]. Serum CysC therefore meets some of the characteristics of an ideal biomarker of true glomerular function [41]. Early studies showed increases in CysC concentrations within 24–36 hours following paraquat poisoning [3,42] which plateaued while creatinine continued to rise [3]. Our much larger study confirms these observations (Fig 2D). However the rise was modest and maximal concentrations of CysC were observed after 24 hours with no further increase seen over the next 3 days (Fig 3). During this period, creatinine rose to > 300% of baseline. In contrast, the relative increase in CysC in AKI in ICU patients is greater and usually earlier than creatinine [43]. In the paraquat-induced AKI patients, the relative increase in creatinine versus CysC increased with time. At face value, this translates into a much greater than two-fold reduction in GFR based on serum creatinine versus cystatin C which is unlikely (Fig 4). Although few non-renal factors tend to increase [44] sCysC levels, reduced serum levels were observed only in hypothyroid states [45]. All patients in in this cohort were previously healthy young adults and hence it is unlikely non-renal factors may have reduced the CysC levels. Increase in urinary cystatin C following paraquat poisoning may be due to altered reuptake and degradation by proximal tubules [46] which confirms paraquat-induced tubular injury.

Creatinine (molecular weight 113 Daltons) is produced by non-enzymatic metabolism of creatine in skeletal muscles. Almost all (>98%) creatine is stored in the skeletal muscles [47,48]. Daily creatine demand is met through diet or de novo synthesis. Initial creatine biosynthesis occurs in the kidney, initiated by enzymatic catalysis of arginine and glycine. The remaining steps that lead to creatine formation and release occur in the liver. Creatine and phosphorylcreatine are utilised to generate ATP in tissues with high energy demands such as skeletal and cardiac muscle and brain [48] and also in oxidative stress. Normally, around 2g of creatine is converted to creatinine by spontaneous non-enzymatic dehydration each day in a 70 kg individual and this creatine must be replaced from diet or by de novo synthesis. Creatinine production is reduced in sepsis due to reduced energy demands [49].

Creatinine is converted to creatol and methylguanidine [50–52] in the presence of free radicals, which may disturb the creatine and creatinine equilibrium and enhance the demand for creatine. Creatinine, creatol and methylguanidine act as scavengers to reduce circulating hydroxyl free radicals [52], and the creatol/creatinine ratio may be used as a marker of oxidative stress after kidney transplantations [51] and in patients with chronic kidney disease (CKD) [50]. Increased production of creatol and other creatinine metabolites have been shown to decrease progression of CKD in rat a model as a result of these anti-oxidant properties[50,52].

We suggest that the abrupt increase in creatinine follows increased production and possibly mobilisation of creatine and creatinine in the kidneys and liver to meet both increased energy demand as a result of oxidative stress and as a homeostatic anti-oxidant scavenging response. Paraquat toxicity is characterized by the induction of oxidative stress with generation of reactive oxygen species, depletion of NADPH, damage to mitochondria, lipid peroxidation, oxidation of proteins, carbohydrates, DNA and sulphide groups [32]. Therefore, the early increase in creatinine may be a biomarker of oxidative stress, while after 24 hours this is progressively a sign of AKI. This unfortunate combination of mechanisms may explain why it is such an accurate prognostic marker for death [3,8].

Acidosis is another common clinical feature of paraquat poisoning and also a good predictor of death [4]. Although spontaneous cyclisation of creatine to creatinine can occur and is pH dependent, it is only likely to contribute to a 5% increase in creatinine production [53].

Human organic cation transporters (hOCT2) play a key role in the proximal tubular secretion of paraquat ions [54,55]. These transporter families mediate the transport of many xenobiotics [56]. Creatinine secretion in the proximal tubule is also mediated by hOCT2 [57,58] and tubular secretion accounts for approximately 10% of creatinine clearance [46]. Thus, competitive or non-competitive inhibition of creatinine transport by paraquat in proximal tubule cells might also cause a minor increase in plasma creatinine, since the study patients had increased levels of urinary paraquat within 24 hours of the insult (Table 2). However, urinary creatinine did not increase during this period.

Major strengths of this study are the prospective multi-centre nature, inclusion of severe poisoning cases from different geographic regions and rapid recruitment of patients within a median time of 4 hours after paraquat ingestion. This study also has limitations including the absence of a gold-standard measure of GFR such as inulin clearance, which is complex and rarely employed in patients with critical conditions, or isotopic methods, which are generally not routinely available in the study regions. There was no measure of the severity of acidosis in these patients, although acidosis has been well-documented following moderate to severe paraquat poisoning [4]. The amount of paraquat ingested (in mg/kg) could not be estimated since patients or their relatives did not identify the brand name of paraquat product ingested. However, paraquat ingestion was confirmed by qualitative urine dithionate test as described and plasma paraquat concentrations and estimated amount ingested are presented in this manuscript. The MDRD formula was used to estimate baseline creatinine in non-survivors, and an MDRD75 is likely to overestimate the baseline creatinine in previously healthy adults [20]. However, the observed degree and rapidity of change was of such a magnitude that a variation in baseline values would not change the interpretation of the observed effects. Urine output data was only limited to 12 AKI patients as frequent urine output measurements are difficult in a highly crowded rural hospital wards. Finally, frequent urine volume measurements and data related to fluid intake could not be collected in this highly crowded rural emergency setting. Therefore, 24 hour urinary creatinine and cystatin C excretion was not estimated.

## Conclusions

Rapid and large increases in creatinine are a common clinical manifestation of severe paraquat poisoning and greatly exceed that predicted by large decreases in GFR. Limited increases in levels of serum cystatin C confirm this interpretation. The best explanation for the observed rapid large increase in creatinine appears to be increased production and mobilisation of creatine and creatinine to meet the increase energy demand resulting from oxidative stress. Paraquat-induced AKI needs to be evaluated using alternative renal biomarkers of function and injury, which are more specific to renal injury under these conditions.
